# Hybrid Nanosystems of Antibiotics with Metal Nanoparticles—Novel Antibacterial Agents

**DOI:** 10.3390/molecules28041603

**Published:** 2023-02-07

**Authors:** Tatyana I. Shabatina, Olga I. Vernaya, Mikhail Y. Melnikov

**Affiliations:** 1 Department of Chemistry, M.V. Lomonosov Moscow State University, 119991 Moscow, Russia; 2 Department of Fundamental Sciences, N.E. Bauman Moscow Technical University, 105005 Moscow, Russia

**Keywords:** nanometals, metal oxide nanoparticles, antibiotics, resistant bacterial strains, hybrid nanocomposites, antibacterial activity, metal-antibiotic complexes, drug delivery

## Abstract

The appearance and increasing number of microorganisms resistant to the action of antibiotics is one of the global problems of the 21st century. Already, the duration of therapeutic treatment and mortality from infectious diseases caused by pathogenic microorganisms have increased significantly over the last few decades. Nanoscale inorganic materials (metals and metal oxides) with antimicrobial potential are a promising solution to this problem. Here we discuss possible mechanisms of pathogenic microorganisms’ resistance to antibiotics, proposed mechanisms of action of inorganic nanoparticles on bacterial cells, and the possibilities and benefits of their combined use with antibacterial drugs. The prospects of using metal and metal oxide nanoparticles as carriers in targeted delivery systems for antibacterial compositions are also discussed.

## 1. Introduction

Antibiotics are medicines that are used to prevent and treat bacterial infections. Since the first patients were treated with antibiotics, numerous lives have been saved. Unfortunately, every year the problem of the appearance of microorganisms resistant to the action of antimicrobial drugs jeopardizes the potential of antibiotics. Antibiotics resistance occurs when a bacterial strain changes and no longer responds to antibacterial drugs, making infections harder to treat and increasing the risk of disease spread, severe illnesses, and death. Almost all antibiotics introduced for medical, veterinary, or agricultural use have encountered the problem of bacterial strains resistant to antibiotics. Penicillin was successful in controlling bacterial infections during World War II. However, in the 1950s, the resistance of bacteria to penicillin became a substantial clinical problem. The new beta-lactam antibiotics that came to replace penicillin also could not avoid this problem. Vancomycin came into clinical practice in 1972. The medicine coped with the problem longer, and it was even believed that bacterial resistance could not be developed to it. However, the resistance of bacteria to vancomycin was reported in coagulase-negative staphylococci in 1979 and 1983 [1].

Since the late 1960s, the pharmaceutical industry has introduced many new antibiotic substances to address the problem of bacteria’s resistance to antibiotics. However, for the new antibacterial preparations, resistant microorganisms arose over time. Multi-resistant pathogenic super-bacteria that are resistant to two or more antibiotics simultaneously have emerged. At the same time, the portfolio of potential antibiotics is starting to dry up [2]. And the time required for the introduction of a new drug into medical practice has become comparable to or exceeds the time required for the emergence and spread of pathogenic resistant strains. Resistance of important bacterial pathogens to common antimicrobial therapies and the emergence of multidrug-resistant bacteria are increasing at an alarming rate. As a result, at the present time, many decades after the first patients were treated using antibiotics, bacterial infections have again become a threat.

In the past decade, various key organizations, including the Infectious Diseases Society of America, the Centers for Disease Control and Prevention, the World Health Organization (WHO), and the World Economic Forum, have declared antibiotic resistance to be a “global public health concern” [3,4].

## 2. Antimicrobial Resistance: Mechanisms of Occurrence, Characterization, and Ways of Reducing

Resistance of microorganisms to antibiotics is a natural phenomenon of bacteria that develops thanks to the evolutionary adaptability to various environments [5]. Bacteria can acquire resistance genes from other related organisms and exhibit intrinsic antibiotic resistance. For example, *Pseudomonas aeruginosa* has a cell membrane with low permeability, and antibiotics of different compositions and chemical structures are unable to penetrate the bacterial membrane and react with intercellular structures. Bacterial strains can also obtain resistance through various gene transformations or by changing the biochemical mechanisms of their reproduction.

Genetic mechanisms can take place in two main ways: through chromosome mutation and extra chromosomal mutation. *Chromosome mutation* is a spontaneous and irreversible type of resistance occurrence that occurs when gene changes are produced by the genomic sequence of bacteria, specifically in the main chromosomes. These changes are transmitted through offspring. The bacteria would begin to replicate and cause pathologies. *Extra chromosomal mutation* occurs in this case when transmission of genetic material is performed through extra chromosomal material: plasmids, transposons, and integrins. Antimicrobial resistance gene transfer mechanisms in this case include transformation, conjugation, and transduction stages [6].

The bacteria have four biochemical mechanisms of microbial resistance against antibiotics, which are focused on inactivating the antibiotic molecules by protecting their chemical active sites. The mechanism of *antibiotic inactivation* involves the enzymatic inactivation or inactivation of antibiotics through the interaction with acetyl, phosphoryl, and adenylyl chemical groups. Thus, acetylation is one of the mechanisms best known for the inactivation of aminoglycosides and chloramphenicol. *Antibiotic excretion mechanisms* are based on the allocation of antimicrobial drugs through the activation of outlet pumps, which are proteins that can eliminate or get rid of a wide variety of antibiotics and drug compounds from the periplasm to the outside of the cell [7,8]. The *permeability of the outer membrane mechanism* includes the generation of changes in the lipid bilayer. This leads to limited penetration of small molecules such as antibiotics. Some bacteria have managed to create biofilms that prevent antibiotics from penetrating the membrane [9]. *The target modification mechanism* takes place when a bacteria can alter the site where the antibiotic molecules can connect with it and thus deactivate the main function of the antimicrobial drugs [10].

In diagnostic laboratories, the values of minimal inhibitory concentrations (MIC) are used to characterize the sensitivity or confirm the resistance of bacterial strains to antibiotics. MIC is the lowest concentration of an antimicrobial drug, which inhibits the visible growth of microorganisms after overnight incubation [11].

The major driver for overcoming antibiotic resistance is considered to be the use, misuse, and overuse of antibiotics in humans and animals. Any use of antibiotics carries the inevitable harm: it complicates the treatment of future patients. Therefore, the main principle of medicine, “to do no harm”, does not work in any case. In most cases, users hoped that the harm of antibiotic resistance could be mitigated by using antibiotics rationally. Further steps that are needed to reduce the development of antibiotic resistance are listed in [12].

- Reducing the doses in cases of antibiotic prescribing when they are misused and overused.
- Proper antibiotic prescribing is based on noticeable differences in selectivity both between classes of drugs and within them.
- Prescribing the doses and duration of antibacterial treatment, considering the possible occurrence of resistance. Unsurprisingly, the selection of mutational resistance is often promoted by prolonged therapy, infection sites, where it is difficult to achieve high drug concentrations, and underdosage.
- Prescribing antibiotic combinations, since this not only prevents the occurrence of resistance but, in some cases, also has synergy potential.
- Improving infection control in hospitals, including good personal hygiene, the use of barrier equipment, appropriate handling and disposal of sharps and clinical waste, and aseptic (sterile) techniques, will reduce the transmission of antibiotic-resistant bacteria.
- Creation of new antibacterial agents.

The last point concerns not only new antibiotic molecules. Researchers are faced with the task of creating a new generation of antibacterial drugs. Such drugs should not cause resistance in pathogenic microorganisms, or for them, the process should be significantly slower. In addition, the creation of new drug delivery systems must meet the requirements of targeted drug molecule delivery and controlled drug molecule release.

## 3. Metal Nanoparticles (NPs) as Antibacterial Agents against Bacteria Resistance to Antibiotic Molecules

**Figure 1:**
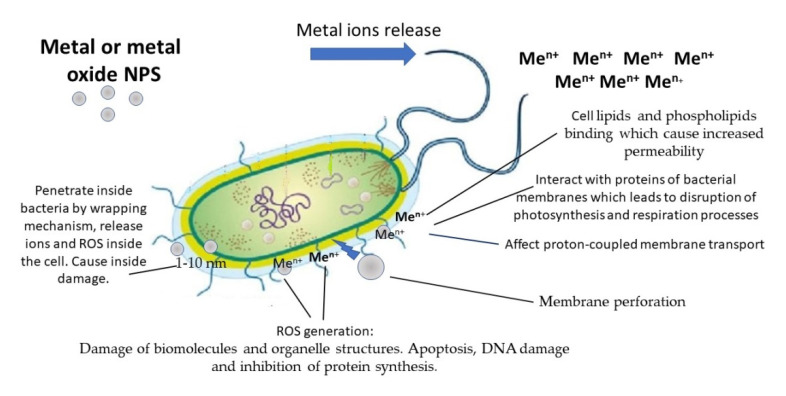
Possible mechanisms of the antibacterial effects of metal and metal oxide nanoparticles (NPs).

The beginning of the 21st century was marked by the rapid development of nanotechnology. This term includes the understanding and control of matter at dimensions between 1 and 100 nm, where unique phenomena enable novel applications [13]. Nanomaterials and nanoobjects have found numerous applications in electronics [14], catalysis [15], photochemistry [16], the paint and lacquer industries [17], ecology [18,19], analytical chemistry [20], and agriculture [21]. Nanotechnologies have also affected medicine and pharmacology [22]. NPs are promising agents for magnetic resonance imaging and hyperthermia, as well as carriers and vectors in targeted drug delivery systems. They are used in clinical assays for magnetic separation and sensing of biological macromolecules and objects [23,24]. The transition to the nanoscale level allowed us to increase the antibacterial activity of several inorganic compounds. NPs of silver, gold, copper, zinc oxide, zirconium, and titanium oxides are considered new antibacterial agents able to solve the problem of the appearance of antibiotic-resistant microorganisms [25,26]. High surface-to-volume ratio of inorganic NPs leads to a multiple increase in various types of interactions with bacterial cells. As a result, nanomaterials can destroy bacterial cells by various mechanisms described below (Figure 1).

### 3.1. Metal and Metal Oxides NPs Mechanisms of Antimicrobial Action

Metal NPs are capable of releasing metal ions both in solution and adsorbed on the surface, and after this, metal NPs can attach to the bacterial cell wall due to electrostatic interactions. Surface metal atoms (surface active centers) of metal or metal oxide NPs can also interact with bacteria due to donor-acceptor interactions [27]. Metal ions have a wide range of chemical and physical properties that define their cell toxicity mechanism. Metal ions may affect multiple targets in the bacterial cell, including enzymes, membranes, and DNA molecules (Figure 1).

Metal ions begin their toxic effects on membranes. Lipids and phospholipids are the main compounds of the bacterial membrane. Both reactive phosphoryl groups in phospholipids and carboxyl groups in unmodified lipids can interact with metal cations. When the ions bind to the cell membrane, the dipole potential of the membrane reduces, resulting in local membrane disruption and an increase in membrane permeability, which finally leads to cell death [27,28]. Copper (Cu^2+^) and silver (Ag^+^) ions interact with proteins of bacterial membranes, and lead to the disruption of biosynthesis and cell respiration processes [29]. Silver NPs were shown to destabilize the outer cell membrane, reduce the plasma membrane potential, and deplete the levels of intracellular adenosine triphosphate molecules [27]. These NPs can affect proton-coupled membrane transport. The type of action of silver NPs was also found to be similar to that of Ag^+^ ions.

The main mechanism by which metal NPs damage bacterial cells is oxidative cell stress caused by reactive oxygen species (ROS). ROS elements include free radicals such as hydroxyl radicals (^•^OH), superoxide radicals (O_2_^•−^), hydroperoxyl (HO_2_^•^), and nonradical species, such as singlet oxygen (O_2_), hydrogen peroxide (H_2_O_2_). ROS elements are significant intermediates of physiological processes, including photosynthesis, respiration, and cell signaling, and their concentration inside the cells is acutely regulated by enzymes [30]. Metal ions induce ROS overexpression, which leads to damage of biomolecules and organelle structures. ROS excess also promotes protein oxidative carbonylation, lipid peroxidation, DNA/RNA breakage, and membrane structure destruction, which cause further cell necrosis, apoptosis, or even mutagenesis [31].

Apoptosis (primarily mitochondrial) has been implicated as a major mechanism of cell death caused by metal NPs-induced oxidative stress [32,33]. High levels of ROS in the mitochondria can result in membrane phospholipid damage and mitochondrial membrane depolarization [34]. Various metal oxide NPs, including zinc, copper, titanium oxides, and silicon dioxide, elicit ROS-mediated cell death via mitochondrial dysfunction [35].

Another frequently reported mechanism is DNA damage and inhibition of protein synthesis. DNA damage may arise due to the formation of Ag^+^-coordinated complexes by Ag^+^ substitution within double and triple hydrogen bonds in DNA base pairs [36]. *Bacillus subtilis* chromosomal DNA degradation takes place in the presence of Ag-NPs. It is assumed that the value of Ag-NPs toxicity is mediated by the concentration of released Ag^+^ ions by Ag-NPs, which can penetrate bacterial cell membranes and subsequently be oxidized intracellularly to Ag_2_O [37]. It is shown that nanosized zinc oxide (ZnO) possesses an antibacterial effect on *Escherichia coli,* which is connected with a great disturbance of the stages of functional gene product synthesis, such as translation, gene expression, RNA modification, and structural constituents of ribosomes [38]. Metal ions can catalyze the oxidation of the susceptible amino acids, impairing protein function, reducing protein stability, and marking the protein for degradation [39].

Less common mechanisms of action for metal and metal oxide NPs are also being proposed. Thus, Ag-NPs with a size of 1–10 nm, according to [40,41], are able to enter inside the bacteria cell and cause its damage due to a direct interaction. The mechanism of the wrapping of metal NPs by membranes and of their penetration into the bacterial cell is proposed in [42]. The experimental studies of the potential key physicochemical properties of metal NPs and their possibility of direct permeation are discussed in [43]. Physical methods such as electroporation and sonoporation for delivering NPs into cells are concidered. Another potential mechanism of metal NPs action on a bacterial cell is cell membrane perforation [44].

Antibacterial mechanisms such as oxidative stress induction, ion release, and disruption of biomolecules are currently well accepted by many researchers. However, the exact antimicrobial mechanisms of the individual metal compounds remain poorly understood.

### 3.2. Ag-NPs

Silver, in all its forms, has been historically used as an antimicrobial agent. Recently, numerous studies have suggested that Ag-NPs exhibit significant antimicrobial actions, specifically against bacterial infections [45,46,47]. Ag-NPs effectively inhibit a wide spectrum of Gram-positive and Gram-negative bacteria, such as *Bacillus cereus*, *Staphylococcus aureus, Micrococcus luteus*, *Streptococcus agalactiae, Escherichia coli, Pseudomonas aeruginosa*, and others [48]. Silver NPs have shown their effectiveness against resistant and multidrug-resistant microorganisms (Table 1).

However, a recent publication devoted to microorganisms-resistant to Ag-NPs [68] showed that gram-negative bacterial strains such as *Escherichia coli* and *Pseudomonas aeruginosa* can develop resistance to Ag-NPs after repeated exposure. The resistance is related to the production of adhesive flagellum protein flagellin, which triggers the aggregation of the NPs. This resistance appears by means of a non-genetic mechanism, and it cannot be overcome by adding additional surfactants or polymers as Ag-NPs stabilizers.

It is likely that the widespread use of silver NPs will lead to the formation of other resistant strains, possibly using genetic mechanisms. Oxidative damage, DNA damage induced by Ag-NPs, and general stress responses can also increase the mutation rate of bacteria [69].

### 3.3. Cu-NPs

Metallic copper (Cu), cupric oxide (CuO), cuprous oxide (Cu_2_O) NPs, as well as their composites of Cu_2_O/CuO, commonly called copper-based NPs, attracted attention due to their antibacterial activity and lower cost compared to Ag-NPs and Au- NPs. Copper ions are shown to be toxic to microbial cells mainly because of the generation of ROS. These NPs exhibit antibacterial activity against numerous bacterial strains, including *Staphylococcus aureus, Staphylococcus epidermidis, Salmonella paratyphi, Klebsiella pneumoniae, Shigella, Pseudomonas aeruginosa, Escherichia coli,* and others [70]. CuO-NPs have been shown to be effective against antibiotic-resistant microorganisms (Table 1). However, it was reported by [71] that both CuO-NPs and copper ions (Cu^2+^) could stimulate the conjugative transfer of multiple-drug resistance genes. This reduces the attractiveness of these particles as antibacterial agents due to their potential hazard when used with antibacterial drugs.

### 3.4. Au-NPs

Au-NPs are considered to be so valuable in the development of antibacterial agents due to their nontoxicity, high ability to functionalize, photothermal activity, and ease of detection. Au-NPs have been widely studied and applied as an effective antibacterial agent against *Staphylococcus epidermidis, Staphylococcus aureus, Bacillus subtilis, Escherichia coli, Pseudomonas aeruginosa, Salmonella typhimurium,* and multiple other bacterial strains [72] including drug-resistant and multidrug-resistant strains (Table 1). Although the generation of ROS is the main cause of cellular death for most antibacterial nanomaterials, the action of Au-NPs does not include ROS. Au-NPs antibacterial activity is implemented in two ways. First step is to inhibit ATPase activities, in order to decrease the ATP level and to collapse the membrane potential. The next step is to inhibit the subunit of the ribosome from binding to tRNA [73]. The ROS-independent mechanisms of action of Au-NPs make them safer for mammalian cells than the other nanometals. The high ability of these NPs for functionalization opens wide prospects for them as targeted antimicrobial agents. However, at the same time, their high cost reduces their attractiveness.

### 3.5. ZnO-NPs and TiO_2_-NPs

ZnO-NPs showed bactericidal effects on gram-positive and gram-negative bacteria [55]: *Staphylococcus aureus, Enterococcus faecalis, Escherichia coli, Pseudomonas aeruginosa*, and *Campylobacter jejuni*. Their putative mechanism of action is related to disruption of the cell membrane and oxidative stress [74,75]. It is also believed that the accumulation of the particles on the bacteria’s surface due to electrostatic forces could be another mechanism of the antibacterial effect of ZnO particles [76].

TiO_2_-NPs possess a large surface area, excellent surface morphology, and are non-toxic in nature. Recently TiO_2_-NPs attracted researchers with their photocatalytic antimicrobial activity, exerting excellent bio-related activity against bacterial contamination [77]. However, oxidative stress, via the generation of ROS, is also proposed for these NPs [26]. TiO_2_-NPs produce ROS under UV light. These particles are also active against antibiotic-resistant microorganisms (Table 1).

## 4. Hybrid Nanosystems “Antibiotic—Metal NPs” and Their Synergetic Antibacterial Effect

Metallic NPs are an effective solution to overcome bacterial resistance to antibiotics [78,79]. However, some of these NPs are toxic, which severely limits their biomedical applications. Recent studies have shown that the combined use of metal NPs with antibiotic drugs can improve their bactericidal effectiveness (Table 2). The revealed bactericidal effect will lead to a reduction in the required doses and a decrease in the toxicity of both agents to human cells. Moreover, the combination of metal NPs and antibiotic drugs will preserve the ability of the latter to destroy bacteria that have become resistant to them.

### 4.1. Ag-NPs

The combined action of Ag-NPs and kanamycin leads to a synergistic increase in bacterial activity. TEM analysis showed that sublethal concentrations of Ag-NPs (6–7 μg mL^−1^) altered the bacterial membrane potential and caused ultrastructural damage, thus increasing the cell membrane permeability. There were no chemical interactions between Ag-NPs and antibiotic drug molecules detected [80].

The antibacterial efficiency of ampicillin, kanamycin, erythromycin, and chloramphenicol against *Staphylococcus aureus, Micrococcus luteus, Escherichia coli,* and *Salmonella typhi* was increased in the presence of Ag-NPs. The authors associate a synergistic increase in antibacterial activity with the bonding reaction between antibiotic molecules and nano-silver. The antibiotic molecules, which contain hydroxyl and amido active groups, can react with Ag-NPs by chelation [81].

Ceftazidime, imipenem, meropenem, and gentamicin sulfate, in combination with Ag-NPs, were tested [82] for their antibacterial effects against three isolates of *Burkholderia pseudomallei*. The results showed that the combination of these antibacterial drugs with Ag-NPs restored antibiotics’ bactericidal efficiency against the bacterial strain that had been shown previously to be resistant to the antibiotics. The bacterial cells were destroyed by the antibiotic–Ag-NPs combinations.

A combination of Ag-NPs and an antibiotic (enoxacin, kanamycin, neomycin, and tetracycline) can synergistically inhibit the bacterial growth of drug-resistant *Salmonella typhimurium* [83]. According to UV–vis and Raman spectroscopy, these four antibiotics can form complexes with Ag-NPs, while ampicillin and penicillin do not. Therefore, no synergistic effect was observed for the latter.

Hybrid systems based on Ag-NPs with the antibacterial drugs dioxidine and gentamicin sulfate have increased antibacterial efficacy (disk diffusion method) against *Staphylococcus aureus, Mycobacterium cyaneum,* and *Escherichia coli* [84]. Their inclusion in biopolymer matrices based on gelatin, calcium alginate, and bovine serum albumin did not lead to the disappearance of the observed effect [85,86].

The combination of Ag-NPs with antibiotics such as polymyxin B or rifampicin showed synergistic antibacterial effects against carbapenem-resistant *Acinetobacter baumannii*. In the case of tigecycline and Ag-NPs, only an additive effect was observed [87]. In vivo with Ag-NPs, the antibiotic combinations led to better survival ratios in *Acinetobacter baumannii*-infected mice than those obtained with single drug treatment.

Ag-NPs (15–25 nm) in combination with antimicrobial agents, including kanamycin, colistin, rifampicin, and vancomycin, displayed synergy against both wild-type and antimicrobial-resistant *Klebsiella pneumonia* isolates [88].

Ag-NPs were effective against the multidrug-resistant bacterial strains *Staphylococcus aureus, Streptococcus pneumoniae,* and *Pseudomonas aeruginosa*. A remarkable reduction in their effective concentration was observed after combination with 1/4 of the MIC of vancomycin [96].

The combination of antibiotic-inorganic NPs makes it possible to cope with microorganisms resistant to antibiotics. Silver covalently bound to cyanographene kills Ag-NPs-resistant bacteria at concentrations 30 times lower than Ag-NPs. The antibacterial activity of the system does not rely on the release of Ag-NPs or ions. Molecular dynamics simulations suggest a strong interaction of Ag-cyanographene with the bacterial membrane [97].

The combined and individual antibacterial activities of the five conventional antibiotics (imipenem, trimethoprim, gentamycin, vancomycin, and ciprofloxacin) and Ag-NPs were investigated against eight different multidrug-resistant bacterial species using the Kirby–Bauer disk-diffusion method. These multidrug-resistant bacterial strains include: *Staphylococcus aureus,* (resistant to trimethoprim and vancomycin); *Micrococcus luteus* (resistant to trimethoprim, gentamycin, and vancomycin); *Enterococcus faecalis*, *Pseudomonas aeruginosa*, and *Escherichia coli* (resistant to trimethoprim, vancomycin, and ciprofloxacin); *Acinetobacter baumannii* (resistant to imipenem, trimethoprim, gentamycin, and vancomycin); *Klebsiella pneumoniae* (resistant to trimethoprim). The synergistic effect of antibiotics and Ag-NPs resulted in a 0.2–7.0 (average, 2.8) fold-area increase in antibacterial activity (Kirby–Bauer disk-diffusion method) [98].

### 4.2. Cu-NPs

Synergistic activity of Cu-NPs with erythromycin, azithromycin, and norfloxacin was detected against Gram-positive bacteria (*Staphylococcus spp*.) and Gram-negative bacteria (*Escherichia coli*, *Klebsiella* spp., *Shigella* spp., and *Pseudomonas* spp.) using the standard disc diffusion method [89].

Cu-NPs with dioxidine hybrid nanocomposites showed enhanced activity compared with the total antibacterial effect of individual components [90].

A synergistic antibacterial effect against *E. coli* was revealed [92] for CuO-NPs combined with cephalexin. It was shown that the presence of antibiotics does not increase Cu^2+^ release, Cu^2+^ uptake, or reactive oxygen species generation. Possible mechanisms of the combined action of the antibiotic molecules and CuO-NPs include the following stages:

- Cephalexin molecules form a high concentration on CuO-NPs surface;
- Concentrated cephalexin molecules interacted more strongly with the *E. coli* cell walls and destroy it more effectively than individual antibiotic molecules;
- CuO-NPs cause secondary damage by inhibiting the lipids and proteins of the cell wall;
- CuO-NPs are easier to get into the cell to bind to the proteins and DNA molecules.

Cu-NPs obtained by means of the green synthesis method using green tea extract (*Camellia sinensis*) were studied for antibacterial activity with antibiotics against *Micrococcus luteus, Streptococcus mutans, Escherichia coli,* and *Salmonella Typhi* [91]. The synergistic activity of Cu-NPs with ampicillin, amoxicillin, gentamicin, and ciprofloxacin was evaluated by means of the disk-diffusion method. It is assumed that the reaction between the antibiotic molecules and Cu-NPs led to synergism. The antibiotic molecules containing the following active groups, such as hydroxyl and amido can easily react with the surface metal centers of Cu-NPs by chelation.

### 4.3. Au-NPs

Synergism between Au-NPs and ceftriaxone against *Klebsiella pneumonia* had been observed [93]. An increase in the antibacterial efficacy of Au-NPs with antibiotics in comparison with antibiotics alone has been established for Au-NPs and gentamicin against *Staphylococcus aureus, Staphylococcus epidermidis* and *Enterococcus faecalis*. This increase was also found for Au-NPs with clindamycin against *Enterococcus faecalis,* Au-NPs with bacitracin against *Staphylococcus epidermidis*, and Au-NPs and polymyxin B against *Staphylococcus saprophyticus*.

Mixture of Au-NPs and cefotaxime demonstrated a synergistic increase in antibacterial activity against *Salmonella typhi, Salmonella typhimurium,* and *Salmonella enteritidis*. However, the combination of Au-NPs with kanamycin exhibited no interaction [94]. The discovered synergism of Au-NPs and antibiotics is associated with the influence of components on the integrity of the membrane.

### 4.4. ZnO-NPs and TiO_2_-NPs

ZnO-NPs conjugated to ciprofloxacin show synergistic antibacterial activity against multiple bacterial pathogens (*Streptococcus* spp., *Bacillus subtilis, Klebsiella* spp., and *Escherichia coli*). There was a 2.9-fold increase in the antibacterial activity of NPs-ciprofloxacin conjugates against *E.coli* and a 2.8-fold increase for *Streptococcus spp*. as compared to ciprofloxacin alone [95].

ZnO-NPs conjugated with clinically approved drugs (quercetin, ceftriaxone, ampicillin, naringin, and amphotericin B) were studied for their activity against several gram-positive (methicillin-resistant *Staphylococcus aureus, Streptococcus pneumoniae,* and *Streptococcus pyogenes*) and gram-negative (*Escherichia coli K1, Serratia marcescens,* and *Pseudomonas aeruginosa*) bacteria. Drug alone and drug-NPs comparisons showed that the NPs exceptionally increased the antibacterial potency of the drugs. Conversely, ZnO-NPs and drug-conjugated NPs showed negligible cytotoxicity against human cell lines except amphotericin B (57% host cell death) and amphotericin B-conjugated with ZnO-NPs (37% host cell death) [99].

However, the joint effectiveness of ZnO-NPs and antibiotics is not always detected. The antimicrobial activity of ZnO-NPs was assessed against pathogenic bacteria (*Escherichia coli)* and fungi (*Aspergillus niger)*. However, in combination with the antibiotic penicillin, there was a decrease in the antimicrobial activity against bacteria and fungi as compared to antibiotics. A possible reason for the decrease in the effectiveness of Zn-O NPs and antibacterial drugs is associated with the use of *Aloe vera* extract for the synthesis of NPs. The presence of this extract may reduce the effectiveness of the interaction of the antibacterial drug with both ZnO-NPs and bacterial cells [100].

Gram-positive and gram-negative bacterial strains’ susceptibility to ZnO-NPs is increasing after the addition of antibiotics. The effect was revealed using the standard microdilution method. Synergistic effects were found for ZnO-NPs with ciprofloxacin, ampicillin, fluconazole, and amphotericin B [101]. Experimental results also demonstrated that doping ZnO-NPs with Fe, Cu, Mn, and Co increases their antibacterial activity, including when used together with antibiotics.

Nanosize TiO_2_ has the enhancement effect on the antibacterial activity of different antibiotics against methicillin-resistant *Staphylococcus aureus* [75]. TiO_2_-NPs and amoxicillin combination has a synergic effect on *Staphylococcus aureus* and *Escherichia coli* growth, as measured by the well diffusion method [102].

Recent studies have shown that NPs can be effectively used in combination with antibiotics, in order to improve their efficacy against various pathogenic microbes.

However, the mechanisms leading to an increase in the effectiveness of hybrid nanosystems including both antibiotics and inorganic nanoparticles, in case of their combination, and the mechanism of activation of antibiotics by metal NPs, are not completely clear. The possible reasons for these effects lie in the fact that nanoparticles, due to their large surface area to volume ratio, present a local high density of antibiotic molecules on the surface to produce polyvalent effects. Inorganic NPs are also shown to act in three sequential stages: membrane destabilization, pore formation, and intracellular fluid leakage [103,104].

Another possible mechanism of action of hybrid nanosystems is based on antibiotics and metal NPs formation of active antibacterial complexes of metal ions and antibacterial drugs. Indeed, such complexes are characterized by increased antibacterial activity and effectiveness against strains resistant to antibiotics [105,106,107]. The formation of the complexes is associated with electron-donor interactions and is due to the presence of nitrogen and oxygen atoms in the chemical structure of drug molecules. For such complexes, it is possible to change the mechanism of action on the bacterial cell compared to the original antibacterial drug.

Both metal ions released from the surface of nanoparticles into the solution and surface atoms (ions) of NPs can participate in the formation of these complexes. In this case, an increase in antibacterial activity is likely with a decrease in the size and an increase in the proportion of surface atoms of inorganic nanoparticles. Also, for particles with a size of less than 10 nm, wrapping mechanisms of penetration into the bacteria are proposed. Such a metal NP could also capture antibiotic molecules.

## 5. Complexes of Antibiotic Molecules and Metal NPs or Metal Ions

**Figure 2:**
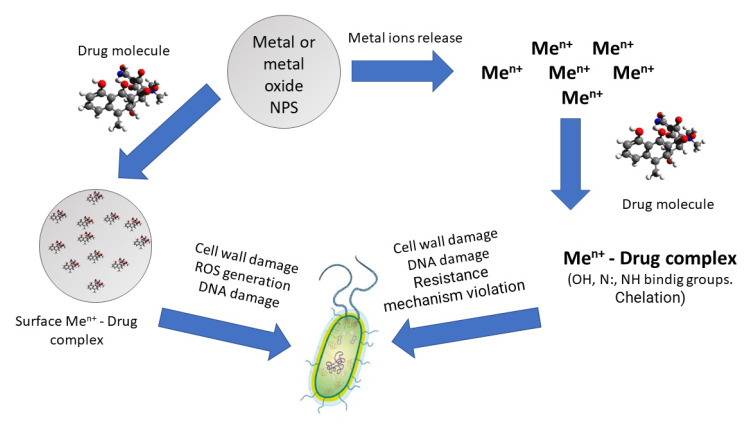
Possible mechanisms of action of complex systems based on antibiotics and the NPs of metals and oxides on bacterial cells.

One of the most proposed and reliable reasons for the synergistic increase in the activity of antibiotics and metal NPs is the formation of “metal atom (metal ion)—drug molecule” complexes. Both metal atoms (surface centers) on the surface of NPs and metal ions in solution that have left the surface of the metal nanoparticle can participate in this process (Figure 2). It is proposed in [108,109] that the coordination of metal atoms/ions with antibiotics is a strategy to reverse resistance and increase their clinical usefulness.

The synergistic effect of Ag-NPs and enoxacin, kanamycin, neomycin, and tetracycline against *Salmonella sp.* revealed in [83] is associated with the discovered NPs-antibiotic complexes. The authors propose a four-stage mechanism. At first, antibiotic molecules form complexes with Ag-NPs. Then the complexes bind to a bacterium. The bacterium-attached antibiotic-Ag-NPs complexes release Ag^+^, more than Ag-NPs alone would release under the same conditions. Thus, a local high Ag^+^ concentration near the surface of the bacterium appears. Finally, Ag^+^ causes bacterial damage, leading to bacterial death. The silver ion toxicity is associated with Ag^+^-ions binding to the proteins and DNA molecules of the cell walls and inside the bacterial cells, disabling the bacterial functions.

The antibacterial activities of ampicillin, kanamycin, erythromycin, and chloramphenicol increased in the presence of Ag-NPs against *Staphylococcus aureus* (gram-positive cocci), *Micrococcus luteus* (gram-positive cocci), *Salmonella typhi* (gram-negative rods), and *Escherichia coli* (gram-negative rods) [81]. It is proposed that the synergistic effect is caused by the bonding reaction between antibiotics’ hydroxyl- and amido-groups and nanosilver by chelation. Ag-NPs became surrounded by antibiotic molecules. In the case of ampicillin, drug molecules act on the cell wall, which leads to cell wall lysis and thus increases the penetration of Ag-NPs into the bacterium. Furthermore, the “Ag-NPs-ampicillin” complex reacts with DNA and prevents DNA unwinding, which results in more serious damage to bacterial cells.

Multidrug-resistant pathogens *Enterobacter spp*. ANT 02 [HM803168], *Pseudomonas aeruginosa* ANT 04 [HM803170], *Klebsiella pneumoniae* ANT 03 [HM803169], and *Escherichia coli* ANT 01 [HM803167] were sensitive to Ag-NPs alone and in combination with ampicillin. All the tested clinical pathogens showed resistance towards ampicillin, either by producing β-lactamases or changing their membrane permeability. All the bacteria were found to be sensitive to Ag-NPs, and the addition of ampicillin slightly enhanced the inhibitory effect on all the tested bacterial strains. The observed effect is associated with the formation of Ag-NPs and ampicillin complexes and their subsequent effect on the bacterial cells. Moreover, each component of the complex has its own impact. The Ag-NPs lyse the cell wall and cause the leakage of internal cellular material, leading to the death of the pathogen. The antibiotic molecules enter the cell through damage caused by Ag-NPs, and that results in irreversible inhibition of the enzyme transpeptidase, which ultimately stops their cell wall synthesis [110].

Synergetic antibacterial activity may be associated with the complexes of metal atoms on the surface of NPs with antibiotic molecules and with metal-ion antibiotic complexes since NPs are able to release ions. Numerous investigations were devoted to such systems. Metal complexes of cefixime (a broad spectrum semi synthetic cephalosporin antibiotic) with Cu(II), Zn(II), Cd(II), Fe(III), and Ni(II) have been synthesized [111]. The Cu(II), Zn(II), Cd(II), and Ni(II) complexes exhibit square planar geometry. The Fe(III) complex exhibits octahedral geometry. All complexes showed higher antimicrobial activity than the cefixime drug only. Among the metal complexes, Fe(III) were more active than other complexes against *Staphylococcus aureus, Escherichia coli, Klebsiella pneumoniae, Proteus vulgaris, Pseudomonas aeruginosa,* and fungal species *Aspergillus niger, Rhizopus stolonifer, Aspergillus flavus, Rhizoctonia bataticola,* and *Candida albicans*.

Ag(I)-camphorimine complexes exert antimicrobial activity on clinically important bacteria *Staphylococcus aureus, Pseudomonas aeruginosa, Burkholderia contaminans,* and *Escherichia coli*. However, none of the complexes were active against *C. albicans* SC5314, which can promote the reduction of the Ag(I) site, forming Ag-NPs as confirmed by SEM [112].

Tetracycline resistance mechanism according [113] is connected with:

1. Ribosomal modification, which prevents drug molecules from binding to it;
2. Converting a drug into an inactive form;
3. Decrease of the membrane permeability;
4. Drug molecules efflux due to the specific pumps.

It is assumed that the drug’s inactive form, which could be easily removed from the cell through the membrane by means of the membrane-associated protein TetA, is the tetracycline-magnesium complex. And a suitable metal complex is not able to be transported Indeed, platinum-tetracycline hydrochloride complex [Pt(C_22_H_24_N_2_O_8_)Cl_2_] was active against tetracyclines-resistant *Escherichia coli*. This complex inhibited unresistant *Escherichia coli* with approximately the same efficiency as tetracycline.

However, the ability to overcome resistance depends on the composition of the tetracycline antibacterial drug. Pt II coordination to oxytetracycline hydrochloride and chlortetracycline hydrochloride does not improve their activity against the tetracycline-resistant *Escherichia coli.* However, the doxycycline hydrochloride complex with Pt II formation made it possible to overcome the resistance of this bacterial strain [114]. The Pd^2+^ complex of tetracycline was 16 times as potent as free tetracycline against the resistant bacterial strain, which expresses Tet-A [115].

## 6. Metal NPs as Carriers in Drug Delivery Systems

Another good reason for including metal NPs in prospective targeted antibacterial drug delivery systems is the possibility to use them as carriers. Metals and NPs can be functionalized according to the characteristics of receptors on the cell surface. These NPs can also be easily modified by polymer covering.

Silver-titanium dioxide NPs covered by polylactic acid (PLA) were designed [116] for norfloxacin (NF) and tenoxicam (TN) targeted delivery. The Ag-NPs fine spots coated with TiO_2_ were collected to form spheres averaging 100 nm in size. It was found that hybrid nanosystems “NF/Ag-TiO_2_/PLA” have an excellent cytotoxic effect against various bacterial cells and tumor cell lines. The speed of drug release from the systems depends on the pH of the surrounding media. The release of drug molecules from the Ag-TiO_2_/PLA systems was faster at pH 7.4. The TiO_2_ hollow spheres with external diameters less than 200 nm and shell thicknesses around 40 nm were obtained by means of a green process [117]. These TiO_2_ hollow spheres were active against gram-positive (*Bacillus subtilis* and *Staphylococcus aureus*) and gram-negative (*Escherichia coli* and *Pseudomonas aeruginosa*) bacterial strains. The possibility of loading the obtained spheres with gentamicin for further use in targeted delivery systems was shown.

Albumin-coated gold nanosystems for antibiotic drugs (tetracycline, oxytetracycline, and rolitetracycline) binding and delivery were obtained [118]. Human serum albumin provides binding sites to drugs and acts as a drug transport vehicle, whereas highly fluorescent property of gold nanoclusters can permit continuous monitoring of the treatment area.

Stronger binding between DNA and hybrid nanosystems “Au-NPs + drugs”, as compared to that of drugs with DNA, was observed. The results suggested that association of the drug molecules with protein or DNA, is strengthened in cases of hybrid “Au-NPs + drugs” systems.

Capped by bovine serum albumin, Au-NPs obtained [119] were functionalized with various amino-glycosidic antibiotics for utilizing them as drug delivery vehicles. The antibiotic conjugated with Au-NP exhibited enhanced antibacterial activity, compared to the pure antibiotic at the same concentration against Gram-positive (*Staphylococcus aureus*) and Gram-negative (*Escherichia coli* and *Pseudomonas aeruginosa*) bacterial strains. It was shown by FTIR that the binding of the antibiotic to the Au-NPs occurs through the amino groups.

Au-NPs functionalized with imipenem or meropenem showed antibacterial activity against carbapenem-resistant gram-negative bacteria isolated from infected humans [120]. Conjugated Au-NPs exhibited a biphasic release profile described as an initial fast release rate that continued for almost 6 h, followed by a steady release phase with a slow release rate that extended up to 72 h.

ZnO:Tb^3+^ hierarchical supramolecular structures synthesized via the solvothermal method and functionalized by organosilicon compounds were used as a carrier for doxorubicin [121]. The systems showed high drug loading and low cytotoxicity.

Hyperbranched polyglycerol-coated Cu-NPs were considered hydrophilic drug delivery carriers for the tetracycline antibiotic. The system provided more than 35% of the drug release through dialysis over five days [122].

Hybrid “Chitosan/TiO_2_-“ and “Chitosan/SiO_2_-“ based drug delivery systems were synthesized by varying, among several parameters, the chitosan molecular weight, the chitosan amount, and the chemical modification of inorganic precursors [110,111,112,113,114,115,116,117,118,119,120,121,122]. Ibuprofen and metoprolol tartrate were chosen as model drugs. The use of optimal synthetic conditions allowed for obtainment of hybrid drug-delivery systems, that are biocompatible, chemically resistant, and possess more than 48 h drug molecules release.

Not only polymer systems and functional groups can serve as binding and retaining components between nanoparticles and antibacterials. The inclusion of dendrimers in a targeted delivery system is one such solution. Pharmadendrimers exhibit antibacterial activity [123] and are capable of becoming not only a connecting link, but also an active component of such systems.

## 7. The Effect of Protein Corona on Antibacterial Targeted Delivery Nanosystems

Targeted drug delivery using nanoparticles can minimize the side effects of conventional pharmaceutical antibacterial agents and increase their effectiveness. However, the introduction of nanoparticle-based antibacterial agents into clinical use still remains a challenge, due to difficulties in regulating interactions at interfaces between nanoparticles and biological systems [124].

When creating antibacterial nanosystems and nanocomposites, it is necessary to take into account that nanomaterials in biological media interact with all occurring biomolecules and form a protein “biocorona”. The biocorona is a very dynamic structure, and its composition changes over time. Biocorona generation affects the effectiveness of the nanopreparation, the “accuracy” of targeted delivery, and it also directs the actions of innate and adaptive immunity. Understanding the process of corona formation is critical to predicting the behavior of NPs in biological systems, including nanotoxicology applications and the development of drug delivery platforms at the nanoscale.

The formation of a protein crown should depend on the physical and chemical characteristics of both NPs and their biological environment [125]. “Corona” formation can be influenced by the nanoparticles used by varying such factors as their constituent components, size, presence of functional groups, coatings, charge, hydrophilicity/hydrophobicity, etc. [126,127].

For unwanted protein uptake, mechanisms can be introduced to prevent or control the composition of the protein corona. One of these mechanisms is to give the NP surface certain functions by including various chemical groups that “hide” it from the “view” of immune cells [128]. A second similar mechanism would be the coating of the NP surface with polymers such as polyethylene glycol (PEGylation), in order to prevent recognition of the NP by the reticuloendothelial system.

However, the protein crown should not always be seen as an artifact to be avoided. In the case of carbohydrate-decorated amphiphilic nanoparticles, a protein crown is formed in the biological environment based mainly on human serum albumin, complement proteins, apolipoproteins, and proteins involved in the coagulation cascade. Although the presence of these protein crowns significantly reduced the cellular uptake of amphiphilic assemblies, they also significantly reduced the cytotoxic and hemolytic effects resulting from the contact of NPs with living cells [129]. The ability to control protein corona formation with ligand molecules has been used to regulate the intracellular activation of nanoenzymes through endosomal proteolysis of coronal proteins [130]. Nanoparticles modified with transition metal catalysts (nanozymes) were created to generate both “hard” irreversible and “soft” reversible coronas in serum. The hard corona induced nanozyme aggregation, effectively inhibiting nanozyme activity. However, only a modest loss of activity was observed with the nonaggregating soft corona nanozymes [131].

Additional research focusing on nanoparticle surface design and the use of orthogonal chemistry will further elucidate the dynamic process of protein biocrown formation, its composition, and the potential for future nanomedicine. Using these results, combined with new drug developments involving NPs, could lead to innovative functional nanotherapies.

## 8. Conclusions

**Figure 3:**
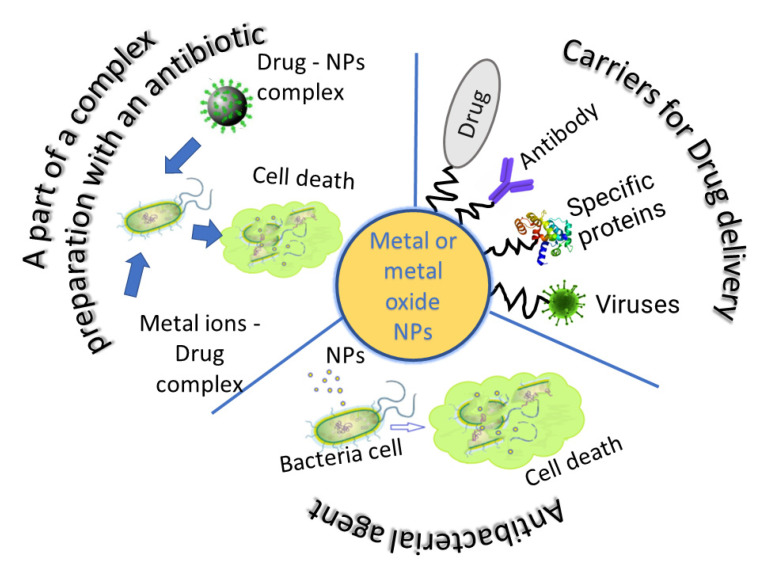
Prospects for the use of metal and oxide NPs as components of antibacterial drugs.

Recent studies have shown that one of the most promising solutions to the resistant microorganisms’ problem lies in the use of metal and metal oxide NPs. It is assumed that, unlike antibiotics, they act on the bacterial cell through several mechanisms, thus reducing the possibility and rate of emergence of resistance to them. The combination of these NPs and antibacterial drug molecules allows not only to expand the range of bactericidal action of hybrid antibacterial systems, since the combination of antibiotics and NPs allows the activity of antibacterial drugs to return, but also, for these hybrid nanosystems, the effect of a synergistic increase in antibacterial activity is manifested. The combination assay provides reduction in therapeutic doses, dose-related toxicity, development of drug resistance, and treatment duration (Figure 3).

The high specific surface area of inorganic particles, together with their ability to be easily modified by binding molecules and polymer coatings, makes metal and metal oxide NPs not only active agents but also promising carriers in targeted drug delivery systems.

## Figures and Tables

**Table 1:** Studies dedicated to the application of metal and metal oxides NPs against drug-resistant bacteria.

NPs, Size (nm)	Synthesis	Bacteria	Antibiotic (or Class) to Which the Microorganism Is Resistant	Method and Concentrations	Reference
Ag					
4–50	Microorganism *Sinomonas mesophila*	*Staphylococcus aureus*	penicillin, methicillin, oxacillin, and gentamycin	Disk diffusion method, 1.56 g Ag/1000 mL	[49]
5–20	Silver nitrate and cyclodextrin	*Pseudomonas aeruginosa*	gentamycin, levofloxacin, piperacillin/tazobactam, cefepime, ceftazidime, ceftriaxone, cefotaxime, and meropenem	MIC range of 1.406–5.625 µg/mL	[50]
7–30	Microorganism *Murraya koenigii* (L.)	*Staphylococcus aureus*	methicillin	Disk diffusion method, MIC 64 μg/ml	[51]
100	Commercially manufactured	*Streptococcus pyogenes,* *Pseudomonas aeruginosa,* and *Escherichia coli O157:H7*	multidrug ampicillin erythromycin	Disk diffusion method	[52]
	Microorganism *Bacillus megaterium*	*Streptococcus pneumoniae,* and *Salmonella typhi*	multidrug multidrug	Disk diffusion method	[53]
20–30	Commercially manufactured	*Pseudomonas aeruginosa*	carbapenem, cephalosporin, aminoglycoside, and fluoroquinolone	Disk diffusion method	[54]
55–83	Green synthesis, extract of *Mimusops elengi*	*Micrococcus luteus,* *Staphylococcus aureus,* and *Klebsiella pneumoniae*	multidrug multidrug multidrug	Disk diffusion method, 5 μg, 10 μg and 15 μg	[55]
4–6	Silver nitrate and sodium hydroxide (60 °C)	*Staphylococcus aureus* and *Escherichia coli*	multidrug multidrug	MIC 40 μg/mL	[56]
5–10	Silver nitrate and exopolysaccharide	*Pseudomonas aeruginosa* and *Klebsiella pneumoniae*	multidrug multidrug	Disk diffusion method (2 mg/mL), MIC 56 μg/ml	[57]
36	Green synthesis, extract of *Tinospora cordifolia*	*Pseudomonas aeruginosa*	amikacin, aztreonam, ceftizoxime, cefepime, gentamicin, imipenem, netilmicin, ofloxacin, piperacillin, and tazobactam	Disk diffusion method (10–100 μg/mL)	[58]
5–40	Fungus *Macrophomina phaseolina*	*Escherichia coli (DH5* *α* *)* *Agrobacterium tumefaciens*	ampicillin and chloramphenicol rifampicin and kanamycin	Disk diffusion assay 5–50 μg/ml	[59]
CuO					
62	Green synthesis, extract of *Momordica charantia*	*Staphylococcus aureus, Streptococcus mutans, Streptococcus pyogenes, Streptococcus viridans, Staphylococcus epidermidis, Corynebacterium xerosis, Bacillus cereus, Escherichia coli, Klebsiella pneumonia, Pseudomonas aeruginosa,* and *Proteus vulgaris*	multidrug	Well diffusion method, concentration of CuO NRs 1.25 mg/50 µL DMSO	[60]
25–30	commercially manufactured Sigma Aldrich	*Staphylococcus aureus, Staphylococcus epidermidis,* and *Enterococcus faecalis*	methicillin methicillin vancomycin	Disk diffusion method	[61]
Au					
3	Egg white, HAuCl4, NaOH	*Staphylococcus aureus*	methicillin	Inhibition zone method, broth microdilution method, MIC 128 μg/mL	[62]
4	BSA, HAuCl_4_, NaOH	*Escherichia coli*	ampicillin, piperacillin, ciprofloxacin, cefotaxime, chloramphenicol, gentamicin, tetracycline, levofloxacin, aztreonam, ceftazidime, cefazolin, piperacillin, tobramycin, oxacillin, and clindamycin	MIC 1–4 μg/mL	[63]
6	HAuCl_4_ with indole or its derivatives	*Escherichia coli,* *Klebsiella pneumonia,* and *Acinetobacter baumannii*	multidrug polymyxin multidrug polymyxin multidrug	MIC 2 μg/mL 2 μg/mL 4 μg/mL 4 μg/mL 4 μg/mL	[64]
4	HAuCl_4_, glutamic acid, C_3_N_4_	*Staphylococcus epidermidis,* *Staphylococcus aureus,* *Bacillus subtilis,* and *Escherichia coli*	ampicillin ampicillin drug-resistant drug-resistant multidrug	Measuring the optical density at 590–600 nm after incubation	[65]
TiO_2_					
20	sol-gel	*Staphylococcus aureus*	methicillin	Disk diffusion method	[66]
20	-	*Streptococcus pneumoniae*	erythromycin, penicillin G, amoxicillin, vancomycin, and moxifloxacin	Agar-well diffusion method 20–40 μg/mL, MIC 100 μg/mL	[67]

**Table 2:** Antibiotic—Metal NPs antibacterial effect.

NPs	Antibacterial Drug	Antibacterial Effect from Combined Application	Bacteria	References
Ag-NPs	kanamycin chloramphenicol	synergistic additive	*E. coli, S. Typhimurium,* and *S. aureus*	[80]
	ampicillin, kanamycin, erythromycin, and chloramphenicol	synergistic	*Staphylococcus aureus, Micrococcus luteus, Escherichia coli,* and *Salmonella typhi*	[81]
	ceftazidime, imipenem, meropenem, and gentamicin sulfate	restore antibiotics bactericidal efficiency	drug-resistant *Burkholderia pseudomallei*	[82]
	enoxacin, kanamycin, neomycin, and tetracycline	restore antibiotics bactericidal effi-ciency, synergistic	drug-resistant *Salmonella typhimuri*	[83]
	dioxidine	synergistic	*Staphylococcus aureus, Mycobacterium cyaneum,* and *Escherichia coli*	[84,85,86]
	rifampicin tigecycline	synergistic additive	*Acinetobacter baumannii*	[87]
	kanamycin, colistin, rifampicin, and vancomycin	synergistic	*Klebsiella pneumonia*	[88]
Cu-NPs	erythromycin, azithromycin, and norfloxacin	synergistic	*Staphylococcus* spp, *Escherichia coli*, *Klebsiella* spp., *Shigella* spp., and *Pseudomonas* spp.	[89]
	dioxidine	synergistic	*Escherichia coli*	[90]
	ampicillin, amoxicillin, gentamicin, and ciprofloxacin	synergistic	*Micrococcus luteus, Streptococcus mutans, Escherichia coli,* and *Salmonella Typhi*	[91]
CuO	cephalexin	synergistic	*Escherichia coli*	[92]
Au-NPs	ceftriaxone	synergistic	*Klebsiella pneumonia*	[93]
	cefotaxime	synergistic	*Salmonella typhi, Salmonella typhimurium,* and *Salmonella enteritidis*	[94]
ZnO	ciprofloxacin	synergistic	*Streptococcus* spp., *Bacillus subtilis*, *Klebsiella* spp., and *Escherichia coli*	[95]

## Data Availability

Not applicable.
